# Targeted Molecular Treatments in Non-Small Cell Lung Cancer: A Clinical Guide for Oncologists

**DOI:** 10.3390/jcm7080192

**Published:** 2018-07-31

**Authors:** Kim Tam Bui, Wendy A. Cooper, Steven Kao, Michael Boyer

**Affiliations:** 1 Chris O’Brien Lifehouse, 119-143 Missenden Road Camperdown, Camperdown, NSW 2050, Australia; kimtam.bui@lh.org.au (K.T.B.); michael.boyer@lh.org.au (M.B.); 2 Sydney Medical School, University of Sydney, Sydney, NSW 2006, Australia; wendy.cooper@health.nsw.gov.au; 3 School of Medicine, Western Sydney University, Sydney, NSW 2560, Australia; 4 Royal Prince Alfred Hospital, 50 Missenden Road Camperdown, Camperdown, NSW 2050, Australia

**Keywords:** personalized therapy, non-small cell, lung cancer, targeted therapy, mutation, rearrangement

## Abstract

Targeted molecular treatments have changed the way non-small cell lung cancer (NSCLC) is managed. *Epidermal growth factor receptor* (*EGFR*), *anaplastic lymphoma kinase* (*ALK*), *v-raf murine sarcoma viral oncogene homolog B1* (*BRAF*), and *c-ros oncogene 1* (*ROS1*) mutations are now used to guide specific anti-cancer therapies to improve patient outcomes. New targeted molecular treatments are constantly being developed and evaluated as a means to improve efficacy, overcome resistance, or minimise toxicity. This review article summarises the current evidence for the efficacy, resistance mechanisms, and safety of targeted molecular treatments against specific mutations in NSCLC.

## 1. Introduction

Worldwide, lung cancer is the most commonly diagnosed cancer and the leading cause of cancer mortality. It accounted for 1.8 million new cases in 2012 (12.9% of all cancers) and caused 1.6 million deaths (19.4% of all cancers) [1]. Non-small cell lung cancer (NSCLC) accounts for 85% of all lung cancer cases, and adenocarcinoma is the most frequent histological subtype, accounting for nearly 40% of all lung cancer cases [2]. Prior to the use of targeted therapy and immunotherapy, patients with advanced lung cancer had a poor prognosis. Platinum doublet chemotherapy, which was the standard of care for all patients with incurable locally advanced or metastatic NSCLC [3], achieved a response rate of 19%, and a median overall survival of 7.9 months [4]. Modern trials with targeted treatments have resulted in significantly better outcomes, with median overall survival now extending to around, or even beyond, two years in selected populations [5,6,7,8].

Specific mutations have been found to be prevalent in lung adenocarcinomas, some of which are predictors for response to targeted treatment. The most common mutations occur in *kirsten rat sarcoma virus* (*KRAS*, found in 24% of cases), *epidermal growth factor receptor* (*EGFR*, 17%), *anaplastic lymphoma kinase* (*ALK*, 3%), *v-raf murine sarcoma viral oncogene homolog B1* (*BRAF*, 2%), and *c-ros oncogene 1* (*ROS1*, 1–2%) [9,10]. We will explore the current evidence for targeted therapy for different mutations in NSCLC, with the aim of providing clinical guidance for oncologists treating NSCLC.

## 2. *EGFR* Mutation-Positive NSCLC

*EGFR* mutations were first described in lung adenocarcinomas in 2004 [11] and were rapidly recognised as a predictor for response to EGFR tyrosine kinase inhibitors (TKIs). The frequency of mutations in this gene varies based on phenotypic characteristics of patients. They occur more frequently in Asian non-smoking women, with an incidence up to 40% [12]. The most common *EGFR* mutations are exon 19 deletions and exon 21 L858R point mutations [9]. Together, these two mutations account for 90% of all *EGFR* mutations [13]. The remaining *EGFR* mutations consist of a range of rarer mutations (which can either be sensitising or non-sensitising with respect to EGFR TKIs), and include exon 18 insertions, G719X point mutations in exon 18 (1–4%), exon 20 mutations (2–5%), and complex mutations (1–2%) [14,15].

EGFR TKIs have been developed to treat *EGFR* mutation-positive lung cancers, and a list of these are included in Table 1.

### 2.1. Efficacy

Early studies investigated the efficacy of EGFR TKIs in a pre-treated population of unselected patients with advanced NSCLC. The BR.21 trial found a 30% improvement in overall survival in patients treated with erlotinib compared to placebo, with an absolute survival benefit of two months [16]. Other studies in unselected populations have not demonstrated a statistically significant overall survival benefit of using EGFR TKIs when compared to chemotherapy or placebo [17,18,19,20], in combination with chemotherapy [21,22], or as maintenance therapy after chemotherapy [23,24,25,26,27,28]. The role of erlotinib in *EGFR* wild-type NSCLC as maintenance therapy was most recently discounted in a phase III trial when erlotinib as maintenance treatment resulted in a median overall survival of 9.7 months compared to a median overall survival of 9.5 months when erlotinib was used on progression [29].

Although designed as a study to select patients based on phenotypic characteristics (ethnicity and smoking history), the Iressa Pan-Asia Study (IPASS) study was the first to demonstrate differential outcomes for patients treated with an EGFR TKI (gefitinib) based on the presence or absence of an activating *EGFR* mutation. These data were based on a subset analysis of patients, which demonstrated that the benefit of EGFR TKIs was exclusive to patients with an *EGFR* mutation [30]. Subsequently, trials have been performed investigating gefitinib, erlotinib, or icotinib in treatment-naive patients selected for the presence of an activating *EGFR* mutation. The results of these trials are summarised in Table 2. Treatment with an EGFR TKI typically resulted in superior median progression-free survival (PFS) of 9–13 months when compared to platinum doublet chemotherapy, which had median PFS in the range of 4–6 months. Furthermore, the response rates were as high as 83% in patients on an EGFR TKI, compared to 36% in patients who received chemotherapy. Due to crossover between the study arms, none of these trials demonstrated a statistically significant improvement in overall survival, which can extend up to 38 months [5,6,30,31,32,33,34,35,36,37].

The question of whether there are meaningful differences between first-generation TKIs has been addressed in three small studies. In these studies, no significant differences between erlotinib and gefitinib were observed, although there were some differences in the pattern of side effects [47,48,49]. The benefits of EGFR TKIs observed with *EGFR* mutation-positive cancers does not translate to patients who have high *EGFR* expression identified using immunohistochemistry or increased *EGFR* copy number detected by fluorescence in situ hybridization [31,50].

In an effort to improve further outcomes for these patients, second-generation EGFR TKIs have been developed. Afatinib and dacomitinib were both designed to bind covalently to the mutated EGFR protein. Additionally, these agents are pan-HER inhibitors and block activation of other members of the *EGFR*/*HER* family. These agents result in superior PFS compared to chemotherapy in treatment-naïve patients with *EGFR*-mutated tumours [7,39,40,43]. There are only limited data comparing second- and first-generation agents to each other. In a randomised phase II study, Lux Lung 7, afatinib and gefitinib were compared as first-line therapy for treatment-naïve patients. Afatinib was found to have a statistically significant PFS benefit; however, the absolute benefit was small (0.1 months) [41,42] and there seems to be little meaningful efficacy difference between the agents. The recently published overall survival data for the phase III trial of dacomitinib compared to gefitinib did show a statistically significant improvement in median overall survival (34.1 vs. 26.8 months). Despite these results, the clinical use of dacomitinib is likely to be limited by the FLAURA trial (discussed in the next paragraph), especially given the toxicity profile of dacomitinib [44].

Osimertinib, a third-generation irreversible EGFR TKI, has greater efficacy than the first- and second-generation agents. FLAURA, a randomised study comparing first-line osimertinib to erlotinib or gefitinib, showed that, despite similar response rates, patients treated with osimertinib had better PFS (18.9 months vs. 10.2 months). In patients who had stable central nervous system (CNS) metastases at time of trial enrolment, osimertinib also had a superior PFS to the first-generation EGFR TKIs (15.2 months vs. 9.6 months). Overall survival data from this study are currently immature [45]. 

There are no prospective studies investigating the efficacy of EGFR TKIs in patients with uncommon *EGFR* mutations. Observational data with small sample sizes do indicate activity of first-generation EGFR TKIs in some of the rarer *EGFR* mutations; however, the response rates may be lower compared to patients with common *EGFR* mutations [14]. In vitro data has demonstrated that cells with exon 18 mutations had better responses to second-generation EGFR TKIs such as afatinib and neratinib compared to first- or third-generation EGFR TKIs [51]. Ad hoc analyses of trial data showed a greater benefit of afatinib in patients with point mutations and duplications in exons 18–21, with a disease control rate of 84%, median PFS of 10.7 months, and median overall survival of 19 months. Meanwhile, patients who had de novo T790M mutations or exon 20 insertions had lower response rates (15% and 9%, respectively), shorter median PFS (2.9 months and 2.7 months), and shorter overall survival (14.9 months and 9.2 months) [52]. The resistance to EGFR TKIs and the poorer prognosis associated with exon 20 mutations was also seen in a retrospective analysis of 20 patients by Noronha et al. [53]. A phase II study of poziotinib in patients with *EGFR* exon 20 mutant advanced NSCLC is currently recruiting, with early results suggesting activity [54]. Without phase III evidence to support a different approach, EGFR TKIs are still the recommended first-line option for patients with uncommon but activating *EGFR* mutations.

Although several studies have been conducted in the adjuvant setting, only one trial has been completed where patient selection was prospectively based on the presence of an activating *EGFR* mutation. Consequently, interpretation of results is difficult. Based on the available data, EGFR TKIs may improve PFS, though the data for overall survival remains immature [55,56,57]. A phase III trial of adjuvant osimertinib in *EGFR* mutation-positive patients is currently recruiting, with results expected in late 2021 [58]. EGFR TKIs have yet to be implemented into routine clinical practice in this setting.

### 2.2. Resistance

Primary resistance, where the best response achieved is progressive disease, is a relatively rare occurrence, and is noted in 4–10% of *EGFR* mutation-positive NSCLC treated with an EGFR TKI [30,32,33,34,36,39,40,41,43,45]. Acquired resistance, where progressive disease develops after a period of objective response or stability, will eventually occur in all patients treated with an EGFR TKI. The most common resistance mechanisms are the T790M mutation (49–62%), c-*MET* amplification (5–22%), *human epidermal growth factor receptor 2* (*HER2*) amplification (12%), epithelial–mesenchymal transition (20%), and small cell lung cancer transformation (3–14%) [59,60,61,62,63,64].

Continuation of a first- or second-generation EGFR TKI after progression on these agents has not been shown to improve outcomes [65,66]. However, osimertinib has demonstrated activity against the exon 20 T790M mutation, which occurs when threonine at position 790 is replaced by methionine [67]. This substitution leads to increased ATP affinity, cell proliferation and survival, and, ultimately, resistance to first generation TKIs [68,69]. AURA3 compared treatment with osimertinib to standard platinum doublet chemotherapy in patients with secondary *T790M* mutation after progression with a first-generation EGFR TKI. Osimertinib resulted in better PFS (10.1 vs. 4.4 months) and higher response rates (70% vs. 30%). A PFS benefit in patients with stable CNS metastases was also seen with osimertinib (8.5 months vs. 4.2 months) [46].

### 2.3. Toxicity

The adverse events of the first-generation EGFR TKIs are often Grade 1 or Grade 2 and are less likely to cause dose reductions (~20%) or drug discontinuation (<6%) compared to chemotherapy. The most common adverse events of any grade are rash or acne (66–80%), diarrhoea (25–55%) and elevated liver transaminases, particularly of alanine aminotransferase (37–55%). Most of the Grade 3 to Grade 5 adverse events occur in <6% of patients, with the exception of elevated transaminases which can occur in up to 26% of patients. The rates of pneumonitis are uncommon, in order of 1–5% [6,30,33,34,36]. There are subtle differences in the pattern of toxicity between erlotinib and gefitinib. Erlotinib is more likely to cause rash or diarrhoea, while gefitinib is more likely to cause liver function abnormalities [49]. However, these differences have little meaningful clinical impact.

Second-generation EGFR TKIs such as afatinib are more likely to result in adverse events when compared to chemotherapy or first-generation EGFR TKIs. Adverse events that are Grade 3 or higher occur in up to 49% of patients and include severe diarrhoea (up to 15%) and rash (up to 16%) [7,41]. In most studies, these have been successfully managed with dose reductions, and consequently the rate of discontinuation of drug as a result of toxicity is comparable to first-generation EGFR TKIs (6–10%) [41,43].

Meanwhile, osimertinib has been shown to have a better toxicity compared to first-generation EGFR TKIs, with a lower frequency and lower severity of adverse events of rash and transaminase elevations [45].

## 3. *ALK*-Positive NSCLC

*ALK* rearrangements were first identified in 2007 [70]. Although a single rearrangement with *echinoderm microtubule-associated protein-like 4* (*EML4*) was initially identified, it has become apparent that there are several variants based on the location of the rearrangement. These variants may have prognostic significance with differences in outcome noted between them [71].

*ALK* rearrangements are more common in younger patients who have never smoked, or who have a light smoking history [72]. The commonly used ALK TKIs are listed in Table 1, and the pivotal randomised controlled trials for ALK TKIs are listed in Table 3.

### 3.1. Efficacy

Crizotinib was initially developed as a mesenchymal-to-epithelial transition (MET) inhibitor. However, during phase I trials it became apparent that it had substantial activity against *ALK*-rearranged tumours [73]. It was the first ALK TKI to show a clinically significant benefit in *ALK*-positive NSCLC, when it was evaluated in the second-line setting compared to chemotherapy. Patients treated with crizotinib had a median PFS of 7.7 months compared to 3.0 months with chemotherapy [8]. Data from this study resulted in crizotinib becoming a standard treatment in this group of patients.

Subsequently, ALK TKIs have been evaluated in the first-line setting. Crizotinib was compared to standard platinum doublet chemotherapy in the PROFILE 1014 study, with better PFS and higher response rates observed for crizotinib than for chemotherapy [74]. A study with the same design but using ceritinib resulted in similar outcomes [75]. Both crizotinib and ceritinib have shown activity in stable CNS metastases compared to chemotherapy, with median PFS of 9.0 months with crizotinib (vs. 4.0 months) and 10.7 months with ceritinib (vs. 6.7 months) [75,76].

Most recently, alectinib has been compared to crizotinib in the first-line setting in the ALEX study. In this study, alectinib had superior efficacy, with the median PFS not reached in the alectinib arm and 11.1 months in the crizotinib arm. Alectinib does have meaningful CNS activity compared to crizotinib. In patients treated with alectinib, the cumulative rate of CNS metastases was markedly reduced (9.4% vs. 41.4%), and, in patients with known CNS metastases at time of trial enrolment, the response rates were higher (59% vs. 26%) [77].

Ensartinib, a third-generation ALK TKI, had response rates of 66% and a median PFS 9.2 months in an early phase I/II trial. Recruitment is currently ongoing for a first-line phase III trial of ensartinib compared to crizotinib in *ALK*-positive NSCLC [78].

In parallel with trials of EGFR TKIs, these studies have all allowed crossover of patients. Consequently, it has not been possible to demonstrate improvements in overall survival.

### 3.2. Resistance

Acquired resistance usually occurs within the first two years of ALK TKI treatment and can occur due to acquired point mutations in *ALK*, or due to bypass track activation via activation of *EGFR* or amplification of *CKIT*. The most common resistance mutation is L1196M, though there are often multiple resistance mutations that occur concurrently [72].

Alectinib and ceritinib have been studied in phase III trials in the second line setting after resistance to crizotinib. They had superior efficacy when compared to chemotherapy with response rates between 35–40%, a median PFS of approximately 9 months, and evidence of efficacy in patients with known CNS metastases [79,80]. Phase II trials investigating brigatinib and lorlatinib show response rates up to 65% in patients with and without CNS metastases [81,82]. A phase II trial for entrectinib, a pan-tropomyosin receptor kinase (TRK), -ROS1, and -ALK TKI, is currently underway for mutation-positive patients with solid tumours after promising phase I data [83].

Using in vitro data on cell lines, Gainor et al. demonstrated that the activity of second-line therapy is influenced by the specific resistant mutation that occurs, as shown in Table 4 [84]. Sequential ALK TKI can also lead to the development of compound *ALK* mutations that may suggest resistance to specific ALK TKIs [85], thus highlighting the importance of patient selection and treatment sequencing when determining the optimal management pathway for each patient.

### 3.3. Toxicity

The most common toxicities of any grade due to crizotinib or ceritinib are diarrhoea (up to 85%), nausea (up to 69%), vomiting (up to 66%), constipation (up to 43%), and raised liver transaminases (up to 53%). Crizotinib is also associated with visual disorders such as visual impairment, photophobia, or blurred vision (71%), oedema (49%), and upper respiratory tract infections (32%) [74,75]. Alectinib is associated with hyperbilirubinaemia but has lower overall rates of gastrointestinal adverse events compared to the other ALK TKIs [77]. The main toxicities from lorlatinib are hypercholesterolaemia and hypertriglyceridaemia [82].

## 4. *ROS1*-Positive NSCLC

*ROS1* rearrangements are more likely to be present in younger, non-smoking Asian patients [10]. There are no TKIs that have been designed to specifically target *ROS1*. However, in early clinical trials, it became apparent that ALK TKIs had activity in *ROS1*-positive patients. Crizotinib had a response rate of 71.7% and a median PFS of 15.9 months in a phase II trial of 127 patients [87]. Ceritinib had a response rate of 62% and a median PFS of 9.3 months in a phase II trial of 32 patients, though the median PFS improved to 19.3 months in patients who were treatment-naïve [88]. Finally, a phase I trial of lorlatinib included 12 *ROS1*-positive patients and achieved a response rate of 50% with a median PFS of 7 months [82]. Importantly, the ALK inhibitor alectinib, which has a structure that is distinct from the other agents mentioned above, is not active in *ROS1*-mutated tumours [89].

## 5. *BRAF* Mutation-Positive NSCLC

*BRAF V600E* mutations have been noted in several tumour types, most notably melanoma. They may also occur in <5% of NSCLC and are often associated with poor response to platinum-based chemotherapy. The availability of RAF inhibitors has led to their evaluation in NSCLC, although the rarity of the mutation means that the data is limited to phase II trials. Dabrafenib in monotherapy resulted in a response rate of 33%, median PFS of 5.5 months and median overall survival of 12.7 months [90]. In keeping with the experience in melanoma, the addition of the Mitogen-activated protein kinase (MEK) inhibitor trametinib resulted in better outcomes (response rates of 64% and a median PFS of 10.9 months) [91]. These agents have regulatory approval for use in NSCLC from the Food and Drug Administration (FDA) and the European Medicines Agency.

### Toxicity

The most common adverse event from dabrafenib and trametinib was pyrexia, which occurred in 64% of patients. Other toxicities such as nausea, diarrhoea, fatigue, peripheral oedema, vomiting, dry skin, anorexia, and headache each occurred in 25–36% patients [91]. 

## 6. *KRAS* Mutation-Positive NSCLC

*KRAS* mutations are the most common mutation found in NSCLC. They often occur at codon 12, and can rarely occur at codon 13 and 61 [92]. They are mutually exclusive with *EGFR* mutations and *ALK* translocations in almost all cases [93]. *KRAS* mutations are more common in smokers, and also convey a poorer prognosis [94,95].

There are no targeted treatments that have a clinically meaningful benefit in patients with *KRAS* mutations. While MEK inhibitors such as selumetinib and trametinib held promise in early research, benefit could not be demonstrated in larger trials in patients with advanced NSCLC. A phase III trial of second-line selumetinib and docetaxel compared to docetaxel alone showed no difference in PFS (3.9 months vs. 2.8 months) and no difference in median overall survival (8.7 months vs. 7.9 months) [96]. A phase II trial of second-line trametinib compared to docetaxel alone showed no difference in median PFS (12 weeks vs. 11 weeks) with a response rate of 12% in both arms [97].

## 7. Other Mutations in NSCLC

There are other less common mutations that have been investigated as potential drug targets. Mutations in the mesenchymal-to-epithelial transition (*MET*) gene that cause exon 14 skipping occur in 3% of non-squamous NSCLC, and are more likely in older patients [98]. Patients with *MET* mutations who never received MET inhibitor therapy had a poor prognosis (median overall survival 8.1 months), which was worse if there was concurrent *MET* amplification (median overall survival 5.2 months). Treatment with a MET inhibitor extended the median overall survival to 24.6 months [99]. Early trials have suggested an antitumour effect of crizotinib in patients with *MET* exon 14-altered NSCLC, and in patients with *MET* amplification [100,101]. Other MET TKIs, such as capmatinib, tepotinib, salvolitinib, cabozantinib, glesatinib, and merestinib, are currently being investigated for patients with *MET* mutations.

*RET* rearrangements occur in 1–2% of patients with NSCLC [102,103]. Vandetanib, lenvatinib, and cabozantinib, which are multitargeted kinase inhibitors, have demonstrated antitumour effect in *RET*-positive NSCLC in phase II trials with response rates ranging from 16% to 53% [104,105,106,107]. Alectinib has also shown promising pre-clinical evidence against *RET*-positive NSCLC [89].

*HER2* mutations occur in 1–6% of patients with NSCLC [108,109,110] and are more common in never-smokers. Gender or ethnicity did not affect incidence of the *HER2* mutations [109]. Retrospective studies have shown responses to HER2-targeted therapies including trastuzumab, neratinib, afatinib, lapatinib, and trastuzumab emtansine [108,111]. Phase II trials have confirmed anti-cancer activity with trastuzumab emtansine and afatinib in patients with *HER2*-mutated NSCLC [112,113]. A further phase II trial investigating the benefit of afatinib is underway. In *HER2*-amplified NSCLC, phase II trials have shown no clinical benefit of trastuzumab monotherapy, trastuzumab with chemotherapy, trastuzumab emtansine, or pertuzumab [114,115,116,117].

## 8. Conclusions

Targeted molecular therapies have revolutionised the management of advanced NSCLC and have become the international standard of care for patients with driver mutations. Individualised patient care has never been so important. The optimal sequencing of TKIs to provide the best outcomes for our patients is unknown, especially in the immunotherapy era of oncology. An improved understanding of molecular resistance will guide the development of new treatments and assist with decision-making about treatment selection.

## Figures and Tables

**Table 1:** Tyrosine kinase inhibitors (TKIs) in non-small cell lung cancer. EGFR: epidermal growth factor receptor; ALK: anaplastic lymphoma kinase.

	First Generation	Second Generation	Third Generation
**EGFR TKIs**	Gefinitib Erlotinib Icotinib	Afatinib Dacomitinib Neratinib	Osimertinib Poziotinib
**ALK TKIs**	Crizotinib	Ceritinib Alectinib Brigatinib	Lorlatinib Entrectinib Ensartinib

**Table 2:** Pivotal randomised controlled trials of Epidermal Growth Factor Receptor (EGFR) TKIs in patients with Stage IIIB/IV non-small cell lung cancer.

Author, Year Trial Name	Country	Population	Intervention *n*	Control *n*	Median Overall Survival (Months) HR (95% CI)	Median Progression-Free Survival (Months) HR (95% CI)	Response Rate
**First line treatment**							
Mok, 2009 [30,31] IPASS	East Asia	Non-smokers Phase III	Gefitinib 609	Carboplatin and paclitaxel 608	18.6 vs. 17.3 HR 0.91 (0.76 to 1.10)	5.7 vs. 5.8 HR 0.75 (0.65 to 0.85)	43% vs. 32.2%
				Subgroup: EGFR mutant Subgroup: EGFR wildtype	HR 0.78 (0.50 to 1.20), NS HR 1.38 (0.92 to 2.09), NS	HR 0.48 (0.36 to 0.64) HR 2.85 (2.05 to 3.98)	71.2% vs. 47.3% 1.1% vs. 23.5%
Mitsudomi, 2010 [5,32] WJTOG3405	Japan	*EGFR* mutation + Phase III	Gefitinib 86	Cisplatin and docetaxel 86	38.4 vs. 37.3 HR 1.25 (0.88 to 1.78)	9.2 vs. 6.3 HR 0.49 (0.34 to 0.71)	62.1% vs. 32.2%
Maemondo, 2010 [33,38] NEJ002	Japan	*EGFR* mutation + Phase III	Gefitinib 115	Carboplatin & paclitaxel 115	27.7 vs. 26.6 HR 0.89 (0.63 to 1.24), NS	10.8 vs. 5.4 HR 0.32 (0.24 to 0.44)	73.7% vs. 30.7%
Zhou, 2011 [34,35] OPTIMAL/CTONG-0802	China	*EGFR* mutation + Phase III	Erlotinib 82	Carboplatin and gemcitabine 72	22.8 vs. 27.2 HR 1.19 (0.83 to 1.71), NS	13.1 vs. 4.6 HR 0.16 (0.10 to 0.26)	83% vs. 36%
Rosell, 2012 [36] EURTAC	Europe	*EGFR* mutation + Phase III	Erlotinib 86	Cisplatin and docetaxel or gemcitabine 87	19.3 vs. 19.5 HR 1.04 (0.65 to 1.68), NS	9.7 vs. 5.2 HR 0.37 (0.25 to 0.54)	58% vs. 15%
Wu, 2015 [6] ENSURE	Asia	*EGFR* mutation + Phase III	Erlotinib 110	Cisplatin and gemcitabine 107	26.3 vs. 25.5 HR 0.91 (0.63 to 1.31), NS	11.0 vs. 5.5 HR 0.34 (0.22 to 0.51)	62.7% vs. 33.6%
Sequist, 2013 [7,39] LUX-Lung 3	International	*EGFR* mutation + Phase III	Afatinib 230	Cisplatin and pemetrexed 115	28.2 vs. 28.2 HR 0.88 (0.66 to 1.17), NS	11.1 vs. 6.9 HR 0.58 (0.43 to 0.78)	56% vs. 23%
Wu, 2014 [7,40] LUX-Lung 6	Asia	*EGFR* mutation + Phase III	Afatinib 242	Cisplatin and gemcitabine 122	23.1 vs. 23.5 HR 0.93 (0.72–1.22), NS	11 vs. 5.6 HR 0.28 (0.20 to 0.39)	66.9% vs. 23%
Park, 2016 [41] LUX-Lung 7 [42]	International	*EGFR* mutation + Phase II	Afatinib 160	Gefitinib 159	27.9 vs. 24.5 HR 0.86 (0.66 to 1.12), NS	11.0 vs. 10.9 HR 0.73 (0.57 to 0.95)	70% vs. 56%
Wu, 2017 ARCHER 1050 [43,44]	Asia	*EGFR* mutation + Phase III	Dacomitinib 227	Gefitinib 225	34.1 vs. 26.8 HR 0.76 (0.58 to 0.99)	14.7 vs. 9.2 HR 0.59 (0.47 to 0.74)	75% vs. 72%
Shi, 2017 CONVINCE [37]	China	*EGFR* mutation + Phase III	Icotinib 148	Cisplatin and pemetrexed 137	Data immature	11.2 vs. 7.9 HR 0.61 (0.43 to 0.87)	64.8% vs. 33.8%
Soria, 2018 [45] FLAURA	International	*EGFR* mutation + Phase III	Osimertinib 279	Gefitinib or erlotinib 277	Data immature	18.9 vs. 10.2 HR 0.46 (0.37 to 0.57)	80% vs. 76% (NS)
**Second line treatment**							
Mok, 2017 [46] AURA3	International	T790M mutation + PD after EGFR TKI Phase III	Osimertinib 279	Platinum and pemetrexed 140	Data immature	10.1 vs. 4.4 HR 0.30 (0.23 to 0.41)	71% vs. 31%

HR: hazard ratio; NS: not significant; PD: progressive disease. IPASS: Iressa Pan-Asia Study; WJTOG: West Japan Thoracic Oncology Group; NEJ: North East Japan Study Group; CTONG: Chinese Thoracic Oncology Group; EURTAC: European Randomised Trial of Tarceva vs Chemotherapy. All trials used consistent dosing of gefitinib 250 mg, erlotinib 150 mg, afatinib 40 mg daily, dacomitinib 45 mg daily, icotinib 125 mg three times a day, and osimertinib 80 mg daily.

**Table 3:** Pivotal randomised controlled trials of Anaplastic Lymphoma Kinase (ALK) TKIs in patients with Stage IIIB/IV non-small cell lung cancer.

Author, Year Trial Name	Country	Population	Intervention *n*	Control *n*	Median Overall Survival (Months) HR (95% CI)	Median Progression Free Survival (Months) HR (95% CI)	Response Rate
**First line treatment**							
Solomon, 2014 [74] PROFILE 1014	International	*ALK*-positive Phase III	Crizotinib 172	Platinum and pemetrexed 171	NR vs. NR 0.82 (0.54 to 1.26), NS	10.9 vs. 7.0 0.45 (0.35 to 0.60)	74% vs. 45%
Soria, 2017 [75] ASCEND-4	International	*ALK*-positive Phase III	Ceritinib 189	Platinum and pemetrexed 187	NR vs. 26.2 0.73 (0.50 to 1.08), NS	16.6 vs. 8.1 0.55 (0.42 to 0.73)	72.5% vs. 26.7%
Peters, 2017 [77] ALEX	International	*ALK*-positive Phase III	Alectinib 600 mg BD 152	Crizotinib 151	NR vs. NR HR 0.76 (0.48 to 1.20), NS	NR vs. 11.1 0.47 (0.34 to 0.65)	82.9% vs. 75.5%
**Second line treatment**							
Shaw, 2013 [8] PROFILE 1007	International	*ALK*-positive PD after chemotherapy Phase III	Crizotinib 173	Pemetrexed or docetaxel 174	Data immature 20.3 vs. 22.8 1.02 (0.68 to 1.54), NS	7.7 vs. 3.0 0.49 (0.37 to 0.64)	65% vs. 20%
Kim, 2017 [81]	International	*ALK*-positive PD after crizotinib Phase II	Brigatinib 90 mg daily 112	Brigatinib 180 mg daily 110	Not reported	9.2 vs. 15.6 0.55 (0.35 to 0.86)	45% vs. 54%
Hida, 2017 [86] J-ALEX	Japan	*ALK*-positive First line or PD after chemotherapy Phase III	Alectinib 300 mg BD 103	Crizotinib 104	Data immature	NR vs. 10.2 HR 0.34 (0.17 to 0.71)	92% vs. 79%
Shaw, 2017 [79] ASCEND-5	International	*ALK*-positive PD after crizotinib Phase III	Ceritinib 115	Pemetrexed or docetaxel 116	Data immature 1.0 (0.67 to 1.49), NS	5.4 vs. 1.6 HR 0.49 (0.36 to 0.67)	39% vs. 7%
Novello, 2017 [80] ALUR	International	*ALK*-positive PD after crizotinib Phase III	Alectinib 600 mg BD	Pemetrexed or docetaxel	Not reported	9.6 vs. 1.4 0.15 (0.08 to 0.29)	36.1% vs. 11.4%

HR: hazard ratio; NS: not significant; PD: progressive disease. All trials with crizotinib and ceritinib used consistent dosing at 250 mg BD and 750 mg daily, respectively.

**Table 4:** Spectrum of activity of different Anaplastic Lymphoma Kinase (ALK) tyrosine kinase inhibitors for different resistance mutations, as studied by Gainor et al. using in vitro data on cell lines [84].

ALK Mutation	Crizotinib	Ceritinib	Alectinib	Brigatinib	Lorlatinib
V1	S	S	S	S	S
C1156Y	I	S	S	S	S
I1171N	I	S	R	S	S
I1171S	I	S	I	S	S
I1171T	I	S	S	S	S
F1174C	I	S	S	S	S
L1196M	R	S	I	S	S
L1198F	S	I	S	S	S
G1202R	R	I	R	I	S
G1202del	I	I	I	I	S
D1203N	I	S	S	S	S
E1210K	S	S	S	S	S
G1269A	I	S	S	No data	S
D1203N + F1174C	R	R	I	I	I
D1203N + E1210K	I	I	I	I	S

* R: resistant; S: sensitive; I: intermediate.
